# A Proteomic Discovery Study of Cerebrospinal Fluid After Aneurysmal Subarachnoid Hemorrhage

**DOI:** 10.1161/STROKEAHA.125.051215

**Published:** 2025-08-12

**Authors:** Ben Gaastra, Luis Coy, Ardalan Zolnourian, Patrick Holton, Ian Galea, Paul Skipp, Diederik Bulters

**Affiliations:** Clinical Neurosciences, Clinical and Experimental Sciences, Faculty of Medicine (B.G., I.G.), University of Southampton, United Kingdom.; Centre for Proteomic Research, School of Biological Sciences and Institute for Life Sciences (L.C., P.S.), University of Southampton, United Kingdom.; Department of Neurosurgery, Wessex Neurological Centre, University Hospital Southampton, United Kingdom (B.G., A.Z., P.H., D.B.).

**Keywords:** aneurysmal subarachnoid hemorrhage, cerebrospinal fluid, humans, outcome assessment, proteomics

## Abstract

**BACKGROUND:**

Proteomic analysis of cerebrospinal fluid (CSF) has the potential to provide insight into the pathophysiology of aneurysmal subarachnoid hemorrhage (aSAH) and target trials to improve outcome. The aim of this study was to perform a definitive proteomic analysis of CSF following aSAH to identify proteins associated with neurological injury.

**METHODS:**

This was a retrospective proteomic analysis of CSF collected at neurosurgical centers in the United Kingdom between 2013 and 2023 either from external ventricular drain or lumbar puncture on day 7 after aSAH. Adults with confirmed aSAH were included. Exclusions were pregnancy, severe comorbidities, inability to follow-up, and those not expected to survive 24 hours. Proteomic analysis was performed using mass spectrometry to identify CSF proteins differentially expressed between patients with good (modified Rankin Scale score of 0–2) and poor (modified Rankin Scale score of 3–6) outcomes at 6 months following aSAH. Controlling for CSF albumin (a marker of blood-brain interface permeability and volume of hemorrhage), differentially expressed proteins were identified. Differential pathway activity was explored using protein interaction, gene set enrichment and TopMD analyses.

**RESULTS:**

A total of 152 patients were included (101 good and 51 poor outcome), and 4952 unique proteins were identified across all samples. The CSF proteomic profile differed between good and poor outcome individuals as evidenced by clustering of individuals by outcome using topological data analysis. Controlling for CSF albumin 16 intracellular and secreted proteins were differentially expressed between good and poor outcome patients. Two cellular pathways were identified to have differential activity by all 3 pathway analysis approaches: the phosphoinositide 3-kinase-Akt signaling pathway and glycolysis/gluconeogenesis.

**CONCLUSIONS:**

In this study, 16 proteins were differentially expressed between good and poor outcome aSAH patients. The proteomic evidence, both on an individual protein and pathway level highlights that inflammation and oxidative injury are associated with the pathophysiology of neurological injury following aSAH. These results support the exploration of treatments targeting these pathways to improve outcome after aSAH.

Aneurysmal subarachnoid hemorrhage (aSAH) is a rare but devastating form of stroke caused by the spontaneous rupture of a cerebral artery aneurysm on the surface of the brain. The lifetime prevalence is 1 in 1000,^1^ which translates to around 70 000 individuals in the United Kingdom at any timepoint. aSAH is associated with significant morbidity and mortality, with up to 40% of patients dying within the first 30 days of hemorrhage.^2^ Even survivors who achieve independence suffer a wide range of non-visible disabilities which impact quality of life and return to work.^3,4^ In the United Kingdom the socioeconomic burden of aSAH has been estimated at £510 million annually,^5^ and although aSAH only represents 5% of strokes, it is responsible for 25% of quality-adjusted life years lost due to stroke.^6^

At present there is only a single drug, the calcium channel blocker nimodipine, to improve outcome following aSAH, although its effect is small.^7^ Despite multiple clinical trials no other medications have been identified to improve outcome.^8^ This highlights that our understanding of the mechanisms underlying neurological injury, and consequently outcome is incomplete. Neurological injury following aSAH can be considered in 2 phases. Early brain injury occurs within the first 72 hours of hemorrhage and is caused by the rapid rise of intracranial pressure that occurs at ictus. Delayed brain injury occurs in the days to weeks after hemorrhage and is caused by toxic cascades initiated by early brain injury and the presence of blood and its breakdown products within the cerebrospinal fluid (CSF).^9^ The main proposed mechanisms involved include cerebral artery vasospasm, inflammation, oxidative injury, microthrombosis, and cortical spreading depression;^9^ however, they all remain controversial, and there is no agreement on which predominates.

To date, most research has taken a targeted approach to study the pathophysiology of aSAH.^10^ The failure of many attempts to resolve the underlying issues emphasizes the need for novel unbiased methodologies to advance our understanding and drive the development of new treatments. An example of this would be our recent genome-wide association study, which highlighted the potential for unbiased methods to provide novel insight by implicating sphingosine-1-phosphate signaling in outcome after aSAH.^11^ Proteomic analysis is another potential unbiased approach that could be used to provide further information on outcome after aSAH.

As brain tissue is not easily available, and CSF is directly related to the site of pathology it is the biosample of choice for proteomic analysis following aSAH.^12^ The human CSF proteome is large with at least 3379 proteins in normal CSF,^13^ and significant changes have been demonstrated in CSF protein content following aSAH.^14^ A number of candidate studies have identified protein biomarkers within the CSF following aSAH associated with complications including delayed cerebral ischemia^10,15^ and outcome.^16^ Up to this point only a small number of pilot studies of the CSF proteome following aSAH have been conducted. One study of 9 patients versus 7 controls demonstrated elevated levels of high mobility group box 1 within the CSF following aSAH and that these levels correlated with outcome.^12^ A second study using 2-dimensional gel electrophoresis and mass spectrometry (MS) to compare traumatic brain injury and aSAH patients versus controls identified significant differences between case and control proteomes.^17^ A more recent study using matrix-assisted laser desorption/ionization time-of-flight MS in 17 patients with aSAH demonstrated significant differences in the CSF proteome not only between cases and controls but also between good and poor outcome aSAH patients.^18^ A further study of 18 patients analyzed changes in the CSF proteome over time and highlighted erythrolysis and the adaptive macrophage response as upregulated following aSAH.^19^ Finally, a recent study using multitargeted proteomics to compare CSF collected by lumbar puncture (LP) from 6 aSAH patients and 6 controls again demonstrated significant differences between the CSF proteome of cases and controls.^20^ These studies all show the potential of this methodology but are limited by small sample sizes, techniques capturing only a small fraction of the CSF proteome, focus on comparison between cases and controls (with minimal comparison of good versus poor outcome patients), no control for covariates, and by predominantly studying the highly selected group of aSAH patients with external ventricular drains (EVDs).

The aim of this study was to conduct a definitive proteomic study addressing all of these limitations to explore the underlying pathophysiological mechanisms of outcome. To do this, we set out to perform MS-based proteomic profiling to identify CSF proteins differentially expressed between good and poor outcome patients controlling for covariates in a large cohort including those without EVDs.

## Methods

This was a retrospective proteomic analysis of CSF comparing individuals with good and poor outcomes after aSAH. Samples were obtained from 2 studies that received both national and institutional approval: (1) SIS (Studies Into Subarachnoid Hemorrhage): an observational study of individuals with aSAH (REC 12/SC/0666, ERGO 5650), and (2) SAS (SFX-01 After Subarachnoid Hemorrhage): a randomized controlled trial of SFX-01 following aSAH^21^ (REC 16/SC/0019, ERGO 91491). The inclusion criteria for SIS were any patient over 16 years with subarachnoid hemorrhage on CT and a radiologically proven aneurysm. The exclusion criteria were prisoners, pregnancy, individuals not expected to survive 24 hours, and bleeding diathesis or thrombocytopenia. The inclusion criteria for SAS were radiological evidence of spontaneous aSAH, original Fisher grade 3 or 4, age 18 to 80 years, presentation within 48 hours, aneurysm treatment not ruled out, and previously independent functional status. The exclusion criteria were elevated plasma creatinine (≥2.5 mg/dL), bilirubin ≥2-fold upper limit of normal, pregnancy, and follow-up not feasible. All patients were managed in accordance with the National Institute for Health and Care Excellence guidance for aSAH (NG228). Full details of management for SAS patients are available in the study article,^21^ with patients in SIS managed by the same clinical team. Written consent was obtained from all participants (or a consultee) in the study. It is reported in accordance with the Strengthening the Reporting of Observational Studies in Epidemiology statement for case-controlled studies.^22^ Anonymized data not published within this article will be made available by reasonable request from any qualified investigator.

### Samples

CSF samples were collected from adult aSAH patients at day 6 to 8 following onset of hemorrhage, either by EVD, if in situ, or LP if no EVD was in situ. Samples were centrifuged for 10 minutes and the supernatant collected and stored at −80 °C. Individuals were excluded if they had evidence of CSF infection, including ventriculitis, on or before the day of sample collection as assessed by CSF microscopy and culture.

### Sample Preparation for MS

CSF extracts containing 100 μg of protein, as assessed using a Direct Detect spectrometer (Merck), were reduced using dithiothreitol and alkylated with iodoacetamide. Samples were digested with sequencing-grade trypsin overnight, and the peptide products were desalted using OASIS PRiME HLB C18 reverse-phase cleanup plates before elution with 70% acetonitrile/water. Samples were subsequently lyophilized and reconstituted in 0.5% formic acid in water for MS analysis.

### MS and Database Search

Peptide extracts (1 μg on column) in buffer A (0.1% formic acid in water [v/v]) were separated on an Ultimate 3000 RSLC nano system (Thermo Scientific) using a PepMap C18 EASY-Spray LC column, 2 µm particle size, 75 µm×75 cm column (Thermo Scientific), over a 140 min linear gradient of 3% to 25% buffer B (0.1% formic acid in acetonitrile [v/v]) at a flow rate of 250 nL/min. Peptides were introduced using an EASY‐Spray source at 2000 V to a Fusion Tribrid Orbitrap mass spectrometer (Thermo Scientific). The ion transfer tube temperature was set to 275 °C. Full MS spectra were acquired from 300 to 1500 m/z in the Orbitrap at 120 000 resolution using TopSpeed mode at a cycle time of 3 seconds. Peptide ions were isolated using an isolation width of 1.6 amu and trapped at a maximal injection time of 120 ms with an AGC target of 300 000. Higher‐energy collisional dissociation fragmentation was induced at an energy setting of 28 for peptides with a charge state of 2 to 4. Fragments were analyzed in the linear ion trap at 30 000 resolution.

Analysis of raw data was performed using Proteome Discoverer software (Thermo Scientific), and the data processed to generate reduced charge state and deisotoped precursor and associated product ion peak lists. These peak lists were searched against the human SwissProt database (June 2021). A maximum of 1 missed cleavage was allowed for tryptic digestion, and the variable modification was set to contain oxidation of methionine and N-terminal protein acetylation. Carboxyamidomethylation of cysteine was set as a fixed modification. The false discovery rate was estimated with randomized decoy database searches and was filtered to 1% at the protein level.

A control CSF sample was regularly analyzed (up to 20 samples between each control) to ensure consistency of MS output.

### Outcome

Outcome was assessed using the modified Rankin Scale^23^ at 6 months following aSAH. Outcome was dichotomized into good (modified Rankin Scale score of 0–2) and poor (modified Rankin Scale score of 3–6) for analysis.

### Statistical Analysis

Samples with a high number of missing proteins compared with the entire cohort were excluded. Only proteins detected in ≥50% of both the good and poor outcome samples were subsequently analyzed. Protein concentrations were normalized to the median protein concentration for each sample. Batch differences were assessed by principal component analysis (PCA) using a probabilistic PCA method implemented with pcaMethods, which estimates missing data.^24^ Topological data analysis (TDA) using all included proteins was implemented in Python using Keppler Mapper^25,26^ with missing data imputed at the protein level using mean imputation. Good and poor outcome cohorts were compared using the Mann-Whitney *U* test for significance as they were nonparametric in distribution. For the primary analysis, protein differences between the good and poor outcome cohorts were considered significant if they achieved a false discovery rate corrected *P*<5% and absolute log_2_(fold change)>1. Proteins reaching significance were also individually tested for association with outcome using multivariable logistic regression controlling for CSF albumin concentration. Albumin is not synthesized or metabolized in the intrathecal compartment, and consequently CSF albumin is a marker of 2 extrathecal sources of CSF proteins: blood-brain interface (BBI) permeability and blood released into the subarachnoid space at the time of aSAH. BBI permeability and the amount of subarachnoid blood are potential confounding variables influencing both CSF proteomic profile and outcome justifying the inclusion of CSF albumin as a covariate in this analysis. In the SAS study, patients received the drug SFX-01, which may confer protection against inflammatory and oxidative injury. Hence, a sensitivity analysis was performed including CSF albumin concentration and treatment status with SFX-01 in the multivariable logistic regression model. An additional sensitivity analysis controlling for source of CSF (LP versus EVD) in addition to CSF albumin concentration was performed and the impact on proteomic profile was assessed with the same PCA method as above. A final sensitivity analysis was performed including analysis batch in addition to CSF albumin concentration in the multivariable logistic regression model. Missing data were excluded from the primary and logistic regression analyses.

To identify differential pathway activity between good and poor outcome cohorts 3 methodological approaches were used: (1) Search Tool for the Retrieval of Interacting Genes/Proteins^27^ analysis with Kyoto Encyclopedia of Genes and Genomes^28^ pathway mapping for differentially expressed proteins achieving a false discovery rate <5% in the primary analysis. (2) Gene set enrichment analysis of KEGG pathways using an absolute signal-to-noise ranking metric on an individual protein level ([mean_poor outcome_–mean_good outcome)_]/[standard deviation_poor outcome_+standard deviation_good outcome_])^29^ for all proteins included in the primary analysis implemented in clusterProfiler.^30^ Enriched pathways were visualized with Pathview.^31^ (3) TopMD^32^ pathway analysis, which maps differential protein expression topologically onto mechanistically linked proteins utilizing WikiPathways^33^ was performed on all proteins included in the primary analysis.

Unless specified, all analyses were performed using R, version 4.2.2.

## Results

A total of 156 day 6 to 8 CSF samples were identified for inclusion, of which 3 were excluded due to CSF infection on or before the day of sampling. A total of 153 CSF samples underwent proteomic analysis, of which 1 sample was excluded due to a high number of missing proteins leaving 152 samples in the final analysis (81 from the SAS study and 71 from the SIS study). One hundred one samples were classified as good and 51 as poor outcome (see Table 1 for demographics of included individuals).

**Table 1.**
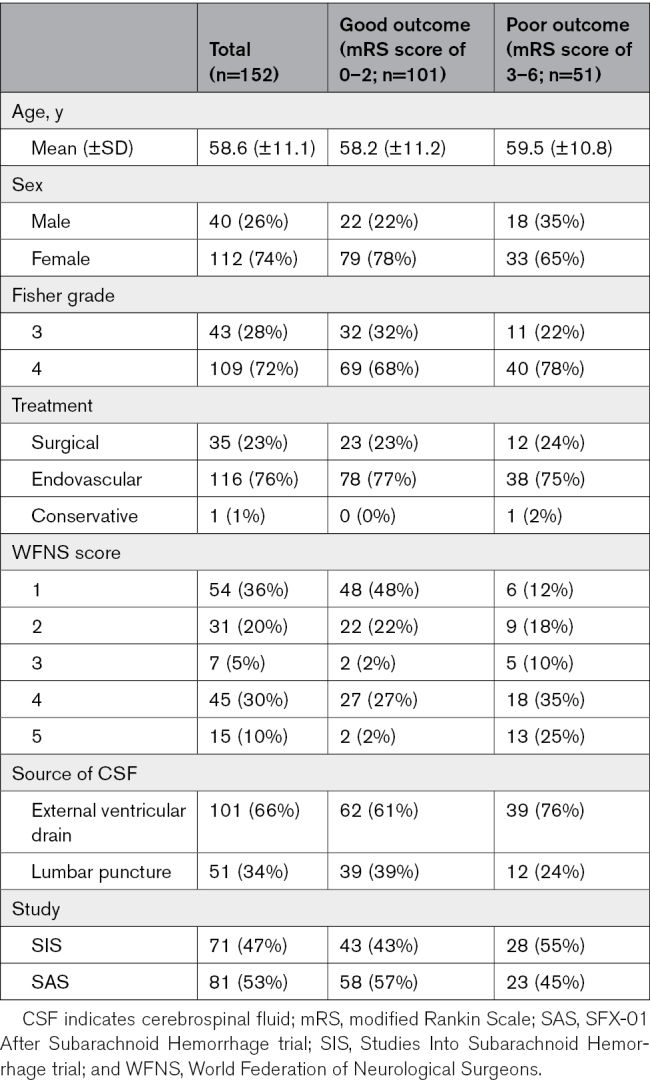
Demographics of Aneurysmal Subarachnoid Hemorrhage Patients. Fisher Grades Are Reported With Reference to the Original Rather Than Modified Rankin Scale

A total of 4952 unique proteins were identified across all samples. The mean number of proteins per sample was significantly higher in the poor outcome compared with the good outcome cohort (poor outcome: 1062 versus good outcome: 950; Wilcoxon rank sum *W*=1967; *P*=0.02). A total of 778 proteins were detected in ≥50% of both the good and poor outcome samples and included in downstream analysis. Proteomic profiling was conducted in 2 batches (corresponding to the 2 studies: SIS and SAS), and PCA demonstrated significant overlap of batches with no requirement for batch correction (Figure S1). The mean of proteins detected in the control sample for the first batch was 797 (±SD 65). In the second batch, using a separate control, the mean number of proteins detected was 973 (±SD 42).

**Figure 1.**
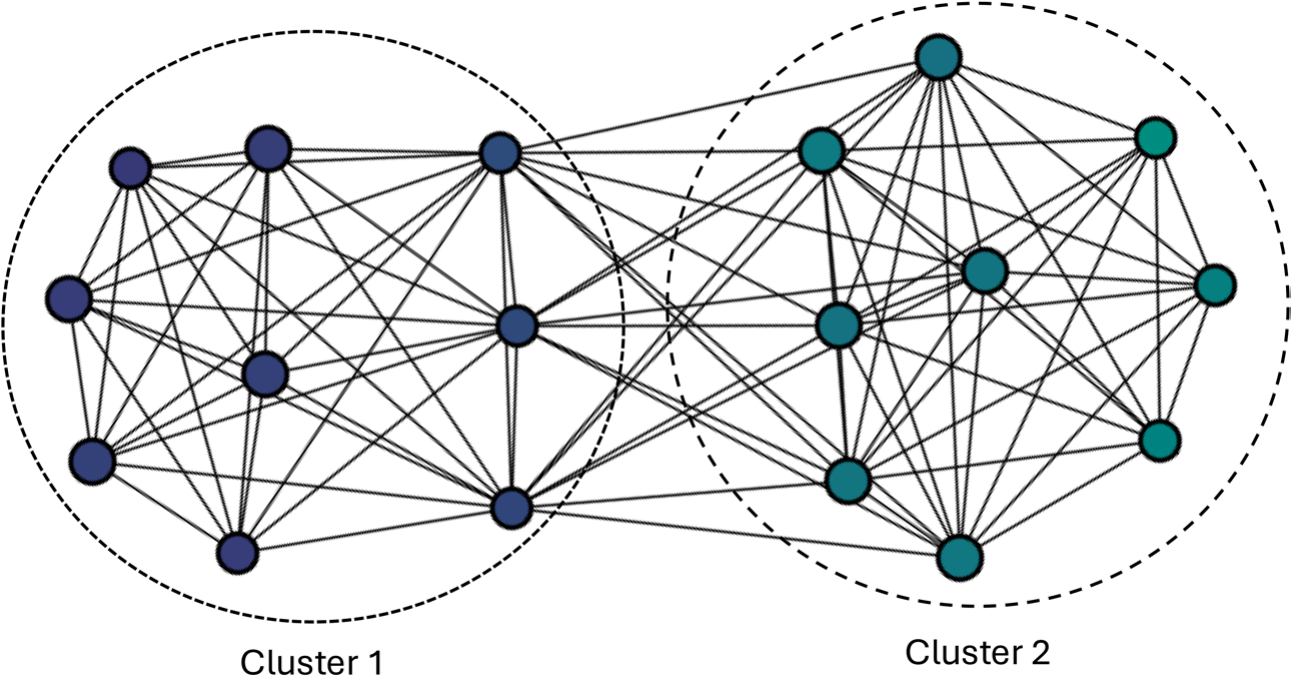
**Topological data analysis using all protein included in primary analysis generated 2 clusters which differ in probability of poor outcome.** Nodes are colored according to probability of poor outcome.

TDA, including all 778 proteins, was performed and optimized using isolation forest and L2 norm lenses, 3 cubes, and allowing up to 70% nodal overlap. TDA grouped samples into 18 nodes. Using the Louvain method for community detection 2 clusters were generated (Figure 1). The probability of poor outcome following aSAH was significantly different between clusters (0.23 versus 0.44; χ^2^=51.8; *P*<0.01).

**Figure 2.**
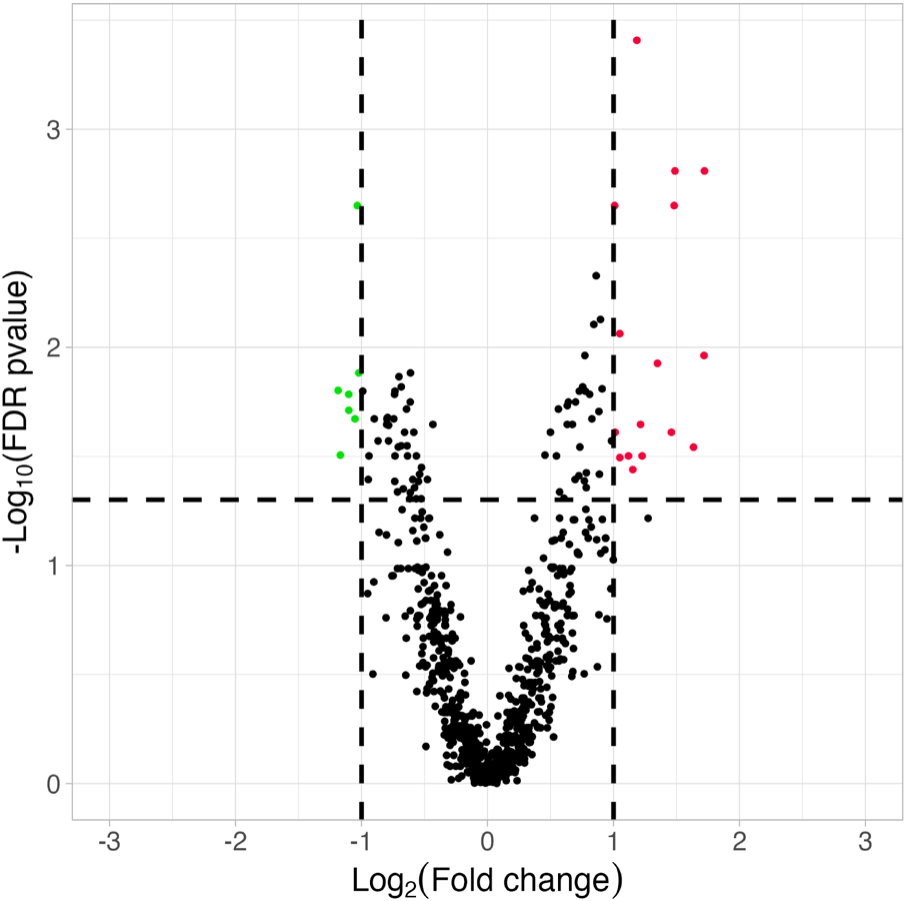
**Volcano plot for primary analysis comparing proteomic differences between individuals with good and poor outcome after aneurysmal subarachnoid hemorrhage.** Red and green dots represent significantly upregulated and downregulated proteins (respectively) in the poor outcome cohort. Dotted lines represent thresholds for significance false discovery rate (FDR) corrected *P*<5% and absolute log_2_(fold change) >1.

In the primary analysis, there was a significant difference in expression of 23 proteins between good and poor outcome cohorts (Figure 2; Table 2). Using logistic regression controlling for CSF albumin concentration, 16 proteins retained significance (Bonferroni correction threshold 0.05/23; Table 2). In a sensitivity analysis including SFX-01 treatment status as an additional covariate in the logistic regression model all proteins which achieved significance after controlling for CSF albumin concentration remained significant. PCA showed significant overlap of proteomic profiles from samples taken from EVD and LP (Figure S2), but 3 proteins: extra cellular matrix protein 2, complement C1q subcomponent subunit A, and actin alpha cardiac muscle 1 did not retain significance in the sensitivity analysis including CSF source as a covariate. When analysis batch was included as a covariate in the logistic regression model, in addition to CSF albumin concentration, all proteins which achieved significance following control for CSF albumin concentration remained significant.

**Table 2.**
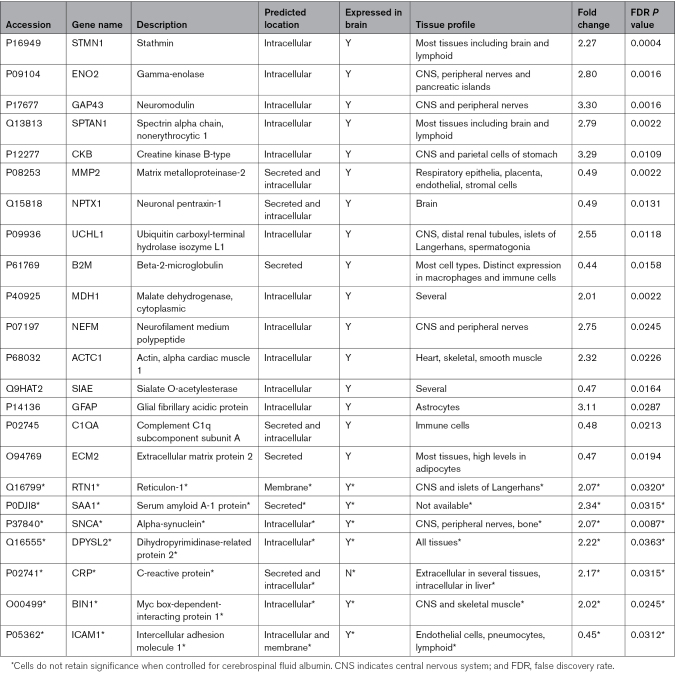
Differentially Expressed Proteins Between Good and Poor Outcome Patients Following Aneurysmal Subarachnoid Hemorrhage. Fold Change Relates to Poor Outcome Cohort

**Figure 3.**
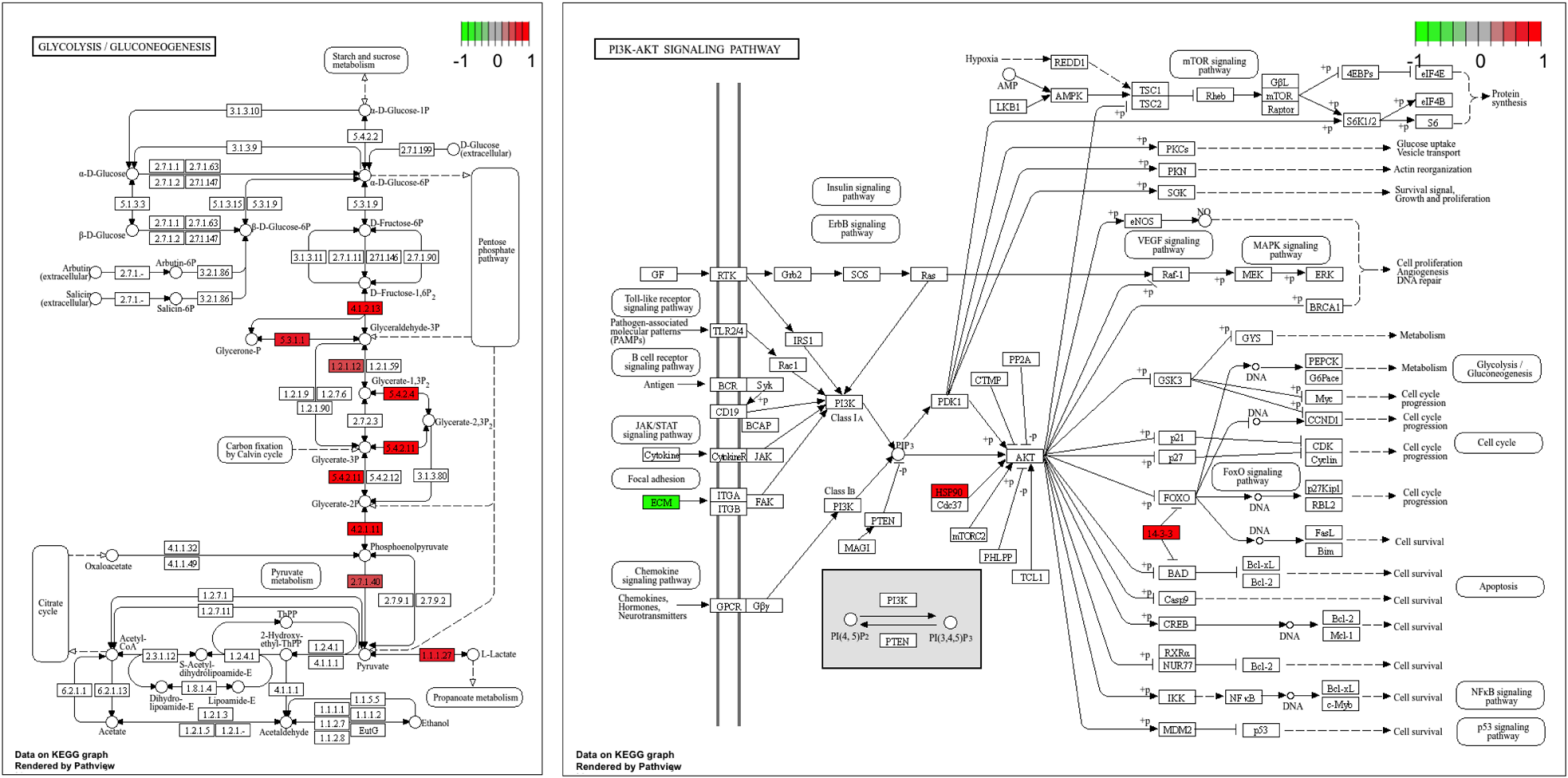
**Glycolysis/gluconeogenesis and phosphoinositide 3-kinase (PI3K)-Akt signaling Kyoto Encyclopedia of Genes and Genomes pathways demonstrating components included in primary analysis (10 proteins from glycolysis/gluconeogenesis and 17 proteins from PI3K-Akt signaling pathways).** Red signifies upregulated and green signifies downregulated in the poor outcome cohort. Relevant enzyme commission components included in the figure are as follows: 4.1.2.13=ALDOA/C; 1.2.1.12=GAPDH; 5.3.1.1=TPI1; 5.4.2.4/11=BPGM; 4.2.1.11=ENO1 & ENO2; 2.7.1.40=PKLR & PKM; 1.1.1.27=LDHB; HSP90=HSP90AA1, HSP90AB1 & HSP90B1; 14-3-3=YWHAQ/B/E/G/H/Z; and ECM=COL1A2, COL4A2, COL6A1/3, FN1, LAMB1/2 & LAMC1.

Two cellular pathways were identified to have differential activity by all pathway analysis approaches (STRING, gene set enrichment analysis, TopMD): the phosphoinositide 3-kinase (PI3K)-Akt signaling pathway (WP4172, hsa04151) and glycolysis/gluconeogenesis (WP534, hsa00010; Figure 3 and Table S1).

## Discussion

In a cohort of 152 individuals, this study demonstrated significant differences between the CSF proteomic profiles of patients with good and poor outcomes after aSAH. Differential protein expression and cellular pathway activity were identified, highlighting PI3K-Akt signaling and oxidative metabolism as key processes involved in the pathophysiology of neurological injury following aSAH.

In this study 4952 unique proteins were identified in CSF following aSAH exceeding the number previously reported in normal CSF.^13^ A greater number of unique proteins were identified in poor versus good outcome aSAH patients, corroborating previous work demonstrating higher CSF protein content in poor outcome patients.^14^

The CSF proteomic profile significantly differed between good and poor outcome individuals as evidenced by clustering of individuals by outcome using TDA. The TDA parameters used in this study allowed up to 70% crossover between nodes, but despite this, only 3 individuals appeared in both clusters emphasizing the ability of proteomic differences to differentiate aSAH patients by outcome. Although the TDA did not perfectly dichotomize individuals by outcome, the probability of poor outcome between the clusters differed almost 2-fold. Overall, this supports the use of CSF proteomic analysis to explore the pathophysiological mechanisms underlying neurological injury and consequently outcome after aSAH.

Twenty-three proteins were differentially expressed between good and poor outcome patients following aSAH, of which 16 retained significance after correction for CSF albumin concentration. All 23 proteins have previously been demonstrated to be present in normal CSF.^13^ Of the 7 proteins previously demonstrated in CSF to be associated with outcome after aSAH (complement C3, histatin 3, angiotensinogen, secretogranin, phospholemman human peptide, serine peptidase inhibitor, and high mobility group box 1),^12,18^ 4 (complement C3, angiotensinogen, secretogranin, high mobility group box 1) were detected in this study although they were either found only in a small number of individuals (high mobility group box 1) or did not achieve *P* value/fold change significance to validate them as biomarkers of outcome. The remaining 3 were detected at different time points to that analyzed in this study, which may explain why they were not identified.^18^

There are 2 potential sources of the differentially expressed proteins in the CSF following aSAH: (1) extrathecal, due to transfer across a permeable BBI or direct entry at time of hemorrhage; and (2) intrathecal, either representing central nervous system cell breakdown or pathophysiological processes taking place following hemorrhage. In this study 16 proteins retain significance after controlling for CSF albumin, indicating that their source following hemorrhage is likely intrathecal; CSF albumin controls for the 2 extrathecal sources of CSF proteins: BBI permeability and bleed size at time of hemorrhage (Table 2). These 16 proteins are all expressed in the brain, further supporting the hypothesis that they are of intrathecal origin. These 16 proteins are intracellular, secreted, or both. Release of intracellular proteins is likely to represent cellular breakdown following hemorrhage meaning these proteins are potential biomarkers of neurological injury following aSAH. Secreted proteins have the potential to provide further insight into the pathophysiological processes taking place within the central nervous system following aSAH. Elevated levels of the intracellular proteins ubiquitin carboxyl-terminal hydrolase isozyme L1 and glial fibrillary acidic protein in plasma and serum, respectively, have been linked to worse outcome after aSAH.^34,35^ We demonstrate their differential levels in the CSF confirming their suitability as biomarkers of outcome after aSAH. The subset of secreted proteins may provide insight into pathophysiology. Elevated levels of serum amyloid A-1 protein in serum have been associated with impaired antiinflammatory function following aSAH.^36^ Matrix metalloproteinase-2 (MMP2) is a secreted protein normally present in CSF and brain tissue and plays a role in neuroinflammation.^37^ Decreased serum MMP2 has been linked to vasospasm following aSAH.^38^ This study replicates these findings in CSF, with serum amyloid A-1 protein elevated and MMP2 decreased in CSF from poor outcome individuals supporting an inflammatory role in neurological injury following aSAH.^9^ On the other hand, elevated serum C1Q is associated with neuroinflammation and has been linked to poor outcome and delayed cerebral ischemia following aSAH^39^ but is not replicated in this study. These proteins were all identified in serum/plasma, and further work is required to assess the interaction with CSF to explore their role in the pathophysiology of aSAH.

Pathway analysis utilizes the breadth of proteomic data generated in this study and has the potential to provide greater insight into the mechanisms driving neurological injury. This study used 3 independent methods to identify differential pathway activity associated with outcome and focused only on pathways highlighted by all 3 methods. This methodological approach is a strength of this study as it cross-validates the highlighted pathways ensuring robust results. The first highlighted pathway was glycolysis/gluconeogenesis. Following aSAH, there is a shift toward hyperglycolysis to provide energy for injured and hypoxic cells.^40^ Abnormal glucose metabolism leading to buildup of metabolites such as pyruvate and lactate has been previously linked to neurological injury and poor outcome in both animals^41^ and humans^42^ following aSAH. Oxidative injury underlies the neurological damage caused by hyperglycolysis and represents a potential therapeutic target, with multiple trials of antioxidants showing benefits in animal models of SAH.^9,43^ Exploration of the components underlying the enrichment of this KEGG pathway suggests there is upregulation of glycolysis and likely indicates a buildup of oxidative metabolites in the poor outcome cohort (Figure 3), in keeping with previous studies. The PI3K-Akt signaling pathway was also highlighted in this study. Activation of this pathway is thought to be neuroprotective with both antioxidant and antiinflammatory effects mediated by downstream signaling pathways such as nuclear factor erythroid 2-related factor 2 and hypoxia-inducible factor 1.^44^ PI3K-Akt signaling has been highlighted as a potential mechanism involved in neurological injury following aSAH.^20^ In animal models of SAH PI3K-Akt activity within the brain is associated with reduced neuronal apoptosis and improved neurological outcome,^45^ whereas pathway inhibition exacerbates neurological injury.^46^ PI3K-Akt signaling has also been linked to neurological recovery following aSAH using overrepresentation analysis of differentially expressed microRNAs between good and poor outcome individuals.^47^ This study supports the importance of PI3K-Akt signaling to outcome after aSAH, although it is not possible to conclude whether overall signaling is up- or downregulated (Figure 3) in this cohort, and further studies are required to confirm its role in the CSF following aSAH. This is particularly important as both upregulation and downregulation of the PI3K-Akt pathway have been associated with neuroprotection in other neurological conditions such as spinal cord injury.^48^

If the threshold for detecting pathways of interest was relaxed to identification by 2 of the 3 analytical methods, then a number of additional pathways are highlighted: biosynthesis of amino acids, estrogen signaling pathway, hypoxia-inducible factor 1 signaling pathway, fluid shear stress and atherosclerosis, and complement and coagulation cascades (Table S1). Notably, no interleukin-based inflammatory pathways were highlighted, although these have been implicated in the pathophysiology of neurological injury after aSAH^49^ and are a target of a recent large randomized controlled trial (URL: https://www.clinicaltrials.gov; Unique identifier: NCT03249207). Although this study does not support the importance of interleukin-based pathways, other approaches such as metabolomics may offer greater sensitivity to highlight these mechanisms of neurological injury.

With a sample size of 152, this is the largest study of the CSF proteome following aSAH. The aim of the study was to explore the associations between the CSF proteome and outcome after aSAH. The approach required for this is not the same as trying to build the most accurate prognostic model for aSAH. We have therefore limited our covariates to confounding variables that influence both the CSF proteome and outcome. Using directed acyclic graph theory, BBI permeability and blood volume released at time of hemorrhage are the only factors identified which fulfill this definition. Therefore, this study has controlled for CSF albumin, which is not synthesized or metabolized in the intrathecal compartment and consequently accounts for these 2 variables. This control for extrathecal proteins is a major strength of the study and allows greater insights to be drawn regarding the pathophysiological processes involved in neurological injury. Other variables are known to influence outcome after aSAH, including delayed cerebral ischemia, and are potential mechanisms by which changes in the CSF proteome may influence outcome. By focusing on whether changes in the CSF proteome influence outcome irrespective of these mediating mechanistic variables, we have positioned this study to identify pathways which influence outcome and could act as potential therapeutic targets. Data are not available on serum albumin levels at day 6 to 8 meaning the serum-CSF albumin concentration ratio cannot be calculated which may more accurately represent BBI permeability due to variation in serum albumin levels. This study is limited to CSF samples collected at a single time point (day 6–8). This timepoint was selected as it is within the peak window of delayed brain injury^8^ and is therefore most likely to provide information on the key processes involved. There are challenges to collecting serial CSF samples from patients not clinically requiring EVDs, and even in analyses limited to patients with CSF diversion it is difficult to achieve sufficient numbers for analysis using unbiased techniques like proteomics as they require multiple comparisons. Nonetheless, future studies should attempt to include serial CSF samples to, first, support the conclusions of this study at different timepoints and, second, assess whether proteomic changes over time are associated with outcome. This study focused on patients with grade 3 and 4 aSAH, as defined by the original Fisher scale. However, with earlier and higher resolution imaging, we found that the vast majority (86%) of consecutively assessed aSAH patients were Fisher grade 3 to 4.^21^ Therefore, although this should be borne in mind when generalizing, this study’s results are applicable to most aSAH patients. Due to the requirement for a CSF sample on day 7, this study did not include patients who died within the first week of aSAH, and caution should be taken when translating the results to this cohort. Although this is a limitation, the aim of this study was to identify mechanisms associated with neurological injury and ultimately target treatments to improve outcome. The cohort of patients who die within the first week of aSAH will have suffered the most severe early brain injury and are likely to be unsalvageable. They are therefore less likely to benefit from a more detailed understanding of the processes involved in delayed brain injury and any potential therapeutic advances. The majority of patients were of European origin, with insufficiently sized subgroups to analyze ethnicity. There is a growing body of evidence that ethnicity may influence both outcome after aSAH and the CSF proteome. Future studies should explore this as a potentially confounding variable. The use of CSF rather than serum/plasma is a major strength of this study, providing insight into pathophysiology as it is closest to the site of pathology, and is therefore more representative. Human brain samples are challenging to collect but proteomic/transcriptomic analyses of brain tissue samples may offer additional information on the processes involved in neurological injury.

This study includes both EVD and LP CSF samples and therefore represents the full spectrum of aSAH patients rather than being limited to the more severe patients with an EVD in situ as in the majority of previous studies. There was significant overlap on PCA, suggesting no influence of CSF source on the majority of proteins (Figure S2). There were 3 proteins that differed between lumbar and ventricular locations (extracellular matrix protein 2, complement C1q subcomponent subunit A, and actin alpha cardiac muscle 1). This is consistent with previous studies which demonstrate differences in the proteomic profile of CSF from the ventricular and lumbar cisterns.^50^ Although it may be more homogeneous to only include samples from a single CSF source, it would significantly impact the generalizability of the study results as it would limit the cohort of individuals included to either EVD or non-EVD participants. Consequently, we have included both EVD and LP CSF samples in this study to maximize generalizability. Future work could take samples from LP regardless of whether an EVD was in situ to address this.

## Conclusions

This unbiased discovery study comparing the proteomic profiles of good and poor outcome aSAH patients demonstrates a greater proteomic diversity in poor outcome individuals and identified 16 proteins differentially expressed between the 2 groups having controlled for CSF albumin. Some were intracellular, like ubiquitin carboxyl-terminal hydrolase isozyme L1 and glial fibrillary acidic protein, and likely represent biomarkers for poor outcome after SAH. However, secreted proteins including MMP2 are more likely to be causal and, alongside multiple methods of pathway analysis implicate inflammatory and oxidative injury as critical pathophysiological mechanisms of neurological injury following aSAH and support exploration of treatments targeting these pathways to improve outcome after aSAH.

## Article Information

### Acknowledgments

Preparation of this article was supported by Dr Gaastra attending the University of Southampton Faculty of Medicine/Faculty of Environmental and Life Sciences Writing Retreat (July 2024).

### Sources of Funding

Dr Gaastra is funded by the National Institute for Health Research (NIHR) and Guarantors of Brain. D. Bulters is funded by the Medical Research Council, NIHR, Rosetrees Trust, and Bio Products Laboratory. Instrumentation in the Centre for Proteomic Research is supported by the Biotechnology and Biological Sciences Research Council, grant/award BM/M012387/1. SIS (Studies Into Subarachnoid Hemorrhage) was funded by the by the Royal College of Surgeons of Edinburgh. SAS (SFX-01 After Subarachnoid Hemorrhage) was funded by Evgen Ltd.

### Disclosures

Dr Skipp discloses shares and consultancy fees in TopMD Precision Medicine Ltd. The other authors report no conflicts.

### Supplemental Material

Table S1

Figures S1 and S2

## Supplementary Material


