# Supplementary material for: A Proteomic Discovery Study of Cerebrospinal Fluid After Aneurysmal Subarachnoid Hemorrhage
Source: Stroke. 2025 Aug 12;56(11):3235–44. doi: 10.1161/STROKEAHA.125.051215 (PMC12551419; doi:10.1161/STROKEAHA.125.051215)
Supplement: Supplementary file 3 [file str-56-3235-s003.pdf]

SUPPLEMENTAL MATERIAL

A proteomic discovery study of cerebrospinal fluid after aneurysmal subarachnoid haemorrhage

Ben Gaastra, Luis Coy, Ardalan Zolnourian, Patrick Holton, Ian Galea, Paul Skipp, Diederik Bulters

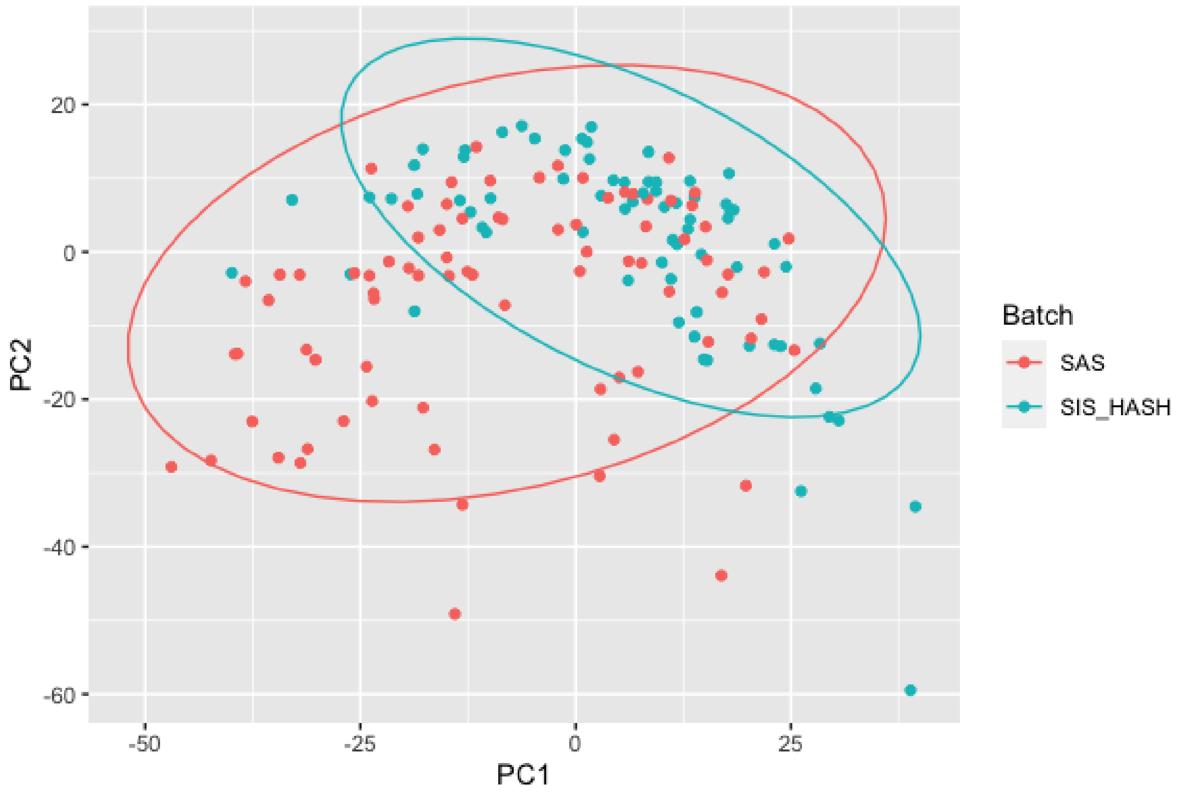

Figure S1. PCA analysis comparing proteomic profiles grouped by batch.

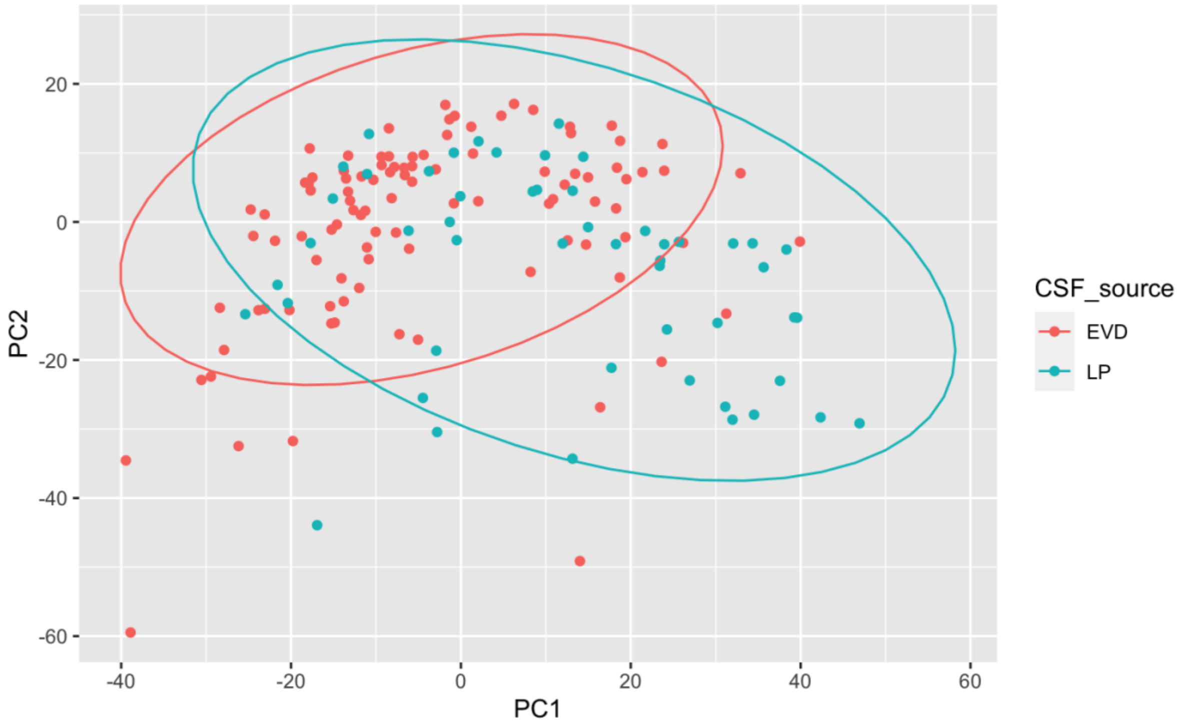

Figure S2. PCA analysis comparing proteomic profiles grouped by CSF source.
